# Carbapenem Resistance in *Acinetobacter baumannii* and Other *Acinetobacter* spp. Causing Neonatal Sepsis: Focus on NDM-1 and Its Linkage to *IS*Aba125

**DOI:** 10.3389/fmicb.2016.01126

**Published:** 2016-08-08

**Authors:** Somdatta Chatterjee, Saswati Datta, Subhasree Roy, Lavanya Ramanan, Anindya Saha, Rajlakshmi Viswanathan, Tapas Som, Sulagna Basu

**Affiliations:** ^1^Division of Bacteriology, National Institute of Cholera and Enteric DiseasesKolkata, India; ^2^Absolut Data Labs, Absolut Data Research and AnalyticsGurgaon, India; ^3^Department of Neonatology, Institute of Postgraduate Medical Education and Research, SSKM HospitalKolkata, India

**Keywords:** *Acinetobacter* spp., ARDRA, NDM-1, OXA-23, Tn125, 5′–3′ CS, neonatal sepsis, India

## Abstract

Carbapenem-resistant determinants and their surrounding genetic structure were studied in *Acinetobacter* spp. from neonatal sepsis cases collected over 7 years at a tertiary care hospital. *Acinetobacter* spp. (*n* = 68) were identified by ARDRA followed by susceptibility tests. Oxacillinases, metallo-β-lactamases (MBLs), extended-spectrum β-lactamases and AmpCs, were detected phenotypically and/or by PCR followed by DNA sequencing. Transconjugants possessing the *bla*_NDM−1_(New Delhi metallo-β-lactamase) underwent further analysis for plasmids, integrons and associated genes. Genetic environment of the carbapenemases were studied by PCR mapping and DNA sequencing. Multivariate logistic regression was used to identify risk factors for sepsis caused by NDM-1-harboring organisms. *A. baumannii* (72%) was the predominant species followed by *A. calcoaceticus* (10%), *A. lwoffii* (6%), *A. nosocomialis* (3%), *A. junni* (3%), *A. variabilis* (3%), *A. haemolyticus* (2%), and 14TU (2%). Fifty six percent of the isolates were meropenem-resistant. Oxacillinases present were OXA-23-like, OXA-58-like and OXA-51-like, predominately in *A. baumannii*. NDM-1 was the dominant MBL (22%) across different *Acinetobacter* spp. Isolates harboring NDM-1 also possessed *bla*_(VIM−2_, _PER−1_, _VEB−2, CTX−M−15)_, *armA, aac(6*′*)Ib, aac(6*′*)Ib-cr* genes. *bla*_NDM−1_was organized in a composite transposon between two copies of *IS*Aba125 in the isolates irrespective of the species. Further, OXA-23-like gene and OXA-58-like genes were linked with *IS*Aba1 and *IS*Aba3 respectively. Isolates were clonally diverse. Integrons were variable in sequence but not associated with carbapenem resistance. Most commonly found genes in the 5′ and 3′conserved segment were aminoglycoside resistance genes (*aadB, aadA2, aac4*′), non-enzymatic chloramphenicol resistance gene (*cmlA1g*) and ADP-ribosylation genes (*arr2, arr3*). Outborn neonates had a significantly higher incidence of sepsis due to NDM-1 harboring isolates than their inborn counterparts. This study demonstrates the significance of both *A. baumannii* and other species of *Acinetobacter* in cases of neonatal sepsis over an extended period. Oxacillinases and *bla*_NDM−1_ are the major contributors to carbapenem resistance. The dissemination of the *bla*_NDM−1_ is likely linked to Tn125 in diverse clones of the isolates.

## Introduction

*Acinetobacter*, a non-fermenting Gram-negative coccobacilli, has been noted as a pathogen in neonatal units in several parts of the globe and outbreaks caused by this organism have also been reported (Huang et al., 2002; Chan et al., 2007; Hammerum et al., 2015). Though *A. baumannii* has been predominantly implicated in neonatal infections or sepsis, “other species” within the genus have also been reported to have caused sepsis in neonates (Beaufort et al., 1999; Kilic et al., 2008). The ability of the species to acquire antibiotic resistance has now resulted in the acquisition of the potent carbapenem resistant gene, *bla*_NDM−1_ (Bonnin et al., 2012; Boulanger et al., 2012; Chuanfu et al., 2013). This enzyme, New Delhi Metallo-β–lactamase-1 (NDM-1), is an addition to the wide array of antimicrobial resistance mechanisms that have been described for *Acinetobacter* spp.

There have been reports of NDM-1 in *Acinetobacter* from different countries (Nordmann et al., 2011). Variants of this enzyme from *Acinetobacter* have also been reported (Espinal et al., 2011). A study has shown that *bla*_NDM−1_ is possibly a chimera that was constructed in *A. baumannii* (Dortet et al., 2014). Though most studies report the presence of this enzyme in *A. baumannii*, NDM-1 has also been detected in other species such as *A. lwoffii* and *A. pittii* (Hu et al., 2012; Pasteran et al., 2014). Apart from NDM-1, carbapenem resistance in *Acinetobacter* is mediated predominantly by the carbapenem-hydrolyzing-oxacillinases belonging to molecular class D (OXA-type carbapenemases) and also by other metallo-β-lactamases (MBLs) belonging to the class B enzymes such as the IMP-and VIM-types.

In addition to the antibiotic resistance, the identification within this genus remains difficult (Vaneechoutte et al., 1995). *Acinetobacter* genus consists of more than 30 species (Turton et al., 2011) and identification requires molecular methods which are not available in the clinical laboratories in developing countries. Thus, these organisms are not identified to the species level using appropriate methods. Until this is done, the association of the various species to nosocomial cases can never be achieved.

Most investigations of *Acinetobacter* in neonatal units have been ad hoc studies that were triggered by an outbreak. There is a dearth of studies that focus on neonatal infections caused by *Acinetobacter* for an extended period of time. In this study we investigate (i) the prevalence of *Acinetobacter* in cases of neonatal sepsis for a period of about 7 years, (ii) the carbapenem-resistant determinants and the genetic context of *bla*_NDM−1_ in *Acinetobacter*, (iii) the transmission of *bla*_NDM−1_ and (iv) association of clinical factors with episodes of sepsis caused due to NDM-1-harboring isolates. The emergence of NDM-1 in this unit makes it relevant to carry out such a study. The use of molecular methods in this study to discriminate the different species of *Acinetobacter* also gives an insight about the different species of *Acinetobacter* as a causative agent of neonatal sepsis.

## Materials and methods

### Setting and patients

The specimens were collected from the neonatal intensive care unit of the IPGMER and SSKM Hospital, Kolkata, India from 2007(January) to 2014(June). Due to some unavoidable circumstances specimens could not be collected from January 2012 to June 2012. This unit has a 16 bed Level III unit, 26 bed Level II unit and 8 bed neonatal surgical units. This unit had 1185 admissions per year (departmental census 2013), including both intramural and extramural births. Blood cultures of the neonates having sepsis were processed by previously described methods (Roy et al., 2013). In brief, 1 ml of blood for culture was drawn with aseptic precautions from a peripheral vein into Peds Plus vials. Blood culture was performed with a BACTEC9050 system (Becton Dickinson, Sparks, MD, USA) from 2008 onwards and manually prior to that. For any culture that flagged positive, Gram-staining was performed and subculture was done on appropriate media based on Gram's stain: MacConkey agar (Difco Laboratories, Detroit, MI, USA) and 5% sheep blood agar (Difco Laboratories) for Gram-negative and Gram-positive organisms, respectively. Clinical data were collected from the hospital registers.

### Identification of *Acinetobacter*

The identity of the *Acinetobacter* spp. was confirmed by VITEK 2 compact system (BioMérieux, Marcy l'Etoile, France) from 2012 onwards and prior to that by the Mini API system (BioMérieux). Molecular identification of all *Acinetobacter* spp. was carried out by the ARDRA (amplified ribosomal DNA restriction analysis) method (Dijkshoorn et al., 1998).

### Antimicrobial susceptibility and MIC

Antimicrobial susceptibility testing was done by the VITEK 2 compact system (BioMérieux) and results were interpreted according to the custom set parameters (CLSI, 2014, FDA guidelines and natural resistance interpretation).

The MIC values (mg/L) of meropenem and ceftazidime were also determined using Etest method (AB Biodisk, Solna, Sweden) and were interpreted according to CLSI (CLSI, 2014). The MIC_50_ and MIC_90_ for meropenem and ceftazidime were calculated as the MIC at which 50 and 90% of the isolates were inhibited.

### Detection of β-lactamase and carbapenemase phenotypes

The production of ESBL, MBL, AmpC was detected by the commercial diagnostic tablets (Rosco Diagnostica A/S, Taastrup, Denmark) following the manufacturer's instructions. The results were interpreted as follows: an increase in ≥5 mm of inhibition zone diameter around boronic acid and dipicoloinic acid, in comparison to the diameter with meropenem alone was considered a positive result for AmpC and MBL respectively, while an increase in ≥5 mm in diameter around clavulanic acid and cloxacillin against cefotaxime alone suggests ESBL and AmpC production respectively (Giske et al., 2010).

### Detection of carbapenemases, AmpCs and ESBLs by genotypic method

PCR was carried out for carbapenem-resistant genes (*bla*_VIM, IMP, SPM−1, GIM−1, SIM−1, NDM_, and *bla*_OXA−23−*like, OXA*−24−*like, OXA*−51−*like, OXA*−58−*like*_) (Ellington et al., 2007; Roy et al., 2011a; Datta et al., 2014) extended-spectrum-β-lactamase genes (*bla*_CTX−M, VEB, PER_) (Cao et al., 2002; Saladin et al., 2002; Colom et al., 2003; Woodford et al., 2006) and *bla*_AmpC_ genes (Perilli et al., 2007) (Table S1).

Class 1 and class 2 integrons (Corvec et al., 2003; Shibata et al., 2003), plasmid-mediated quinolone resistance genes *aac(6*′*)-Ib-cr* and aminoglycoside resistance genes (*rmtA, rmtB, rmtC, rmtD, and armA*) (Berçot et al., 2011) were additionally investigated in the NDM-1-producing isolates (Table S1).

### Conjugation and electro-transformation of *bla*_NDM−1_

Conjugal transfer of *bla*_NDM−1_ to the sodium azide-resistant *E. coli* J53 recipient (Pasteran et al., 2014) was attempted by a solid mating assay (Walsh et al., 2011). Transconjugants were selected with varying concentrations of meropenem (0.1–2 mg/L) and 100 mg/L of sodium azide. Electro-transformation into commercially available MAX Efficiency® DH10B™ Competent Cells (Life Technologies, Carlsbad, CA, USA) was carried out for non-conjugative strains using 0.1 mg/L meropenem.

Susceptibility test for ceftazidime and meropenem was performed for the *bla*_NDM−1_ carrying transconjugants and transformants by Etest method and was followed by PCRs.

### Plasmid characterization

Plasmids harbored by the donor (*bla*_NDM−1_-harboring isolates), transconjugants or transformants were purified by Kado and Liu method, 1981 and were subsequently assessed for the number and sizes (approx) using *E. coli* V517 and *S. flexneri* 2a YSH6000 (Roy et al., 2011b) as mega-plasmid marker (Kado and Liu, 1981).

### Sequencing of the amplified product

Amplified products of *bla*_NDM_, *bla*_VIM_, *bla*_VEB_, *bla*_PER_, *bla*_AmpC_, *bla*_CTX−M_ were sequenced directly on both strands using Big dye Terminator v3.1 Cycle Sequencing Kit and analyzed with an automated sequencer (ABI 3730 DNA Analyzer, Perkin Elmer, USA). The *bla*_*NDM*_ in some isolates could not be directly sequenced and were cloned into a TOPO TA cloning vector (Life technologies) and sequenced with the M13 primers.

### Characterization of integrons

Integrons were amplified and sequenced with primers derived from the 5′ and 3′ conserved segments (Novais et al., 2006) and submitted to the INTEGRALL database (http://integrall.bio.ua.pt) for nomenclature. Some of the large products (>1500 bp) were sequenced by primer walking technique (Sverdlov and Tatyana, 2005).

### Genetic environment of the carbapenem-resistant genes

The genetic structures surrounding the *bla*_NDM−1_, were studied by PCR mapping and DNA sequencing using primers based on previously reported structures (Poirel et al., 2011; Bonnin et al., 2012; Table S1). In the NDM-1-possessing transconjugants and transformants, the proximity of the *IS* elements to genes were determined using combinations of the respective *IS* primers and the OXA-23-like and OXA-58-like primers (Lin et al., 2010).

### Pulsed field gel electrophoresis (PFGE)

PFGE was carried out for all isolates in a CHEF-DR III apparatus (Bio-Rad Laboratories, Hercules and CA) by ApaI enzyme (Abbo et al., 2005) (New England Biolab, Massachusetts).

When indistinguishable isolates of *Acinetobacter* spp. were cultured from two or more neonates treated in the NICU during an interval of up to 15 days, they were considered as a single episode of cross-transmission (Roy et al., 2010).

### Statistical analysis

The data for the 68 neonates along with all their recorded variables (clinical factors, results of tests, organisms found, antibiotic resistance patterns etc.) were entered in IBM SPSS Statistics Version 20.0 and analyzed systematically using established statistical procedures. Characteristics were described as median and standard deviation (SD) for continuous variables and as frequencies and percentages for categorical variables.

Features like sex, gestational age, birth weight, mode of delivery, use of mechanical ventilator and place of birth (intramural or extramural) that are known to be associated with sepsis were analyzed for association with episodes of sepsis caused due to NDM-1-harboring isolates. Multivariate logistic regression was used to identify the risk factors for infection by NDM-1-harboring organisms. All available clinical factors were entered into the regression simultaneously and a backward selection process was used to identify the risk factors. All relationships were evaluated at a 95% significance level.

Chi-square test of independence was carried out to determine the association of sepsis due to NDM-1 with outcome (death or discharge) and onset of sepsis (early or late). Finally, a simple *t*-test of proportions was used to test if the incidence of infection via *Acinetobacter baumannii* or other species was significantly different for each type of antibiotic resistance. All these tests were evaluated at a 95% significance level.

## Result

### Demographics of the neonates

Twenty out of 68 (29.4%) neonates from whom *Acinetobacter* spp. was isolated were female while 46 were male (gender information was missing for 2 neonates). The median gestational age for the neonates was 32.5 weeks (SD 3.4 weeks) and median birth weight was 1363 grams (SD 828 grams). Eighty two percent (56 of 68) of the neonates were pre-term while 77.9% (53 of 68) of the neonates were of low birth weight. Sixty five percent (44 of 68) of the neonates were delivered by cesarean section while the rest had a normal vaginal delivery. Most of the neonates in this unit were born at the facility (inborn)—46 of 68 (67.6%) and the rest were born at other facilities and later transferred to this unit (out born). Sixty two percent of the neonates had early onset sepsis while 31% had late onset of sepsis (time of onset of sepsis could not be recorded with certainty for 5 neonates).

### Identification of *Acinetobacter* spp. by ARDRA

During this period, 68 non-duplicate *Acinetobacter* spp. were isolated from the blood specimens of 68 septicaemic neonates. All isolates were analyzed in this study. The year-wise breakup of the isolates is depicted in Figure S1 indicating the higher prevalence of *Acinetobacter calcoaceticus*-*baumannii* complex (ACB complex) comprising of *A. baumannii, A. calcoaceticus, A. nosocomialis*, and *A. pittii*.

Each of the 68 isolates was unambiguously identified to the species level by ARDRA. The predominant species was *A. baumannii* (72%) followed by *A. calcoaceticus* (10%), *A. lwoffii* (6%), *A. nosocomialis* (3%), *A. junni* (3%), *A. variabilis* (3%), *A. haemolyticus* (2%), and 14TU (2%) (Table 1).

**Table 1:** **Antibiotic susceptibility pattern (by VITEK 2 compact) and genetic determinants of ***Acinetobacter*** spp**.

**Organism (n)/Antimicrobial agent (overall %)**	**No. of resistant isolates (% of the resistant isolates)**											**Genetic determinants (% of isolates)**						
	**CAZ (79.4%)**	**FEP (75%)**	**AZT (85.2%)**	**DOR (50%)**	**IMP (51%)**	**MEM (51%)**	**AN (60%)**	**GEN (70.8%)**	**CIP (83.8%)**	**MIN (1%)**	**TGC (0%)**	***bla*_NDM−1_ (22%)**	***bla*_AmpC_ (51.5%)**	***bla*_VEB−2_ (10%)**	***bla*_oxa−51−like_ (72%)**	***bla*_oxa−23−like_ (48.5%)**	***bla*_oxa−58−like_ (14.7%)**	***bla*_PER−1_ (35.2%)**
1. *Acinetobacter calcoaceticus-baumannii complex (ACB complex)* (58)	79.3%	75.8%	84.4%	55.1%	55.1%	55.1%	63.8%	74.1%	86.2%	2%	0%	20.6%	58.62%	3.04%	84.4%	55.2%	15.5%	38%
a) *A. baumannii*(49)	41	41	46	29	29	29	34	40	45	1	0	6	27	1	49	31	5	15
b) *A. calcoaceticus*(7)	4	2	2	3	3	3	3	3	5	0	0	4	4	−	−	−	3	4
c) *A. nosocomialis* (2)	1	1	1	0	0	0	0	0	0	0	0	2	2	1	−	1	1	1
2. Other species(10) a) *A. lwoffii*(4)	80% 4	70% 4	90% 4	20% 0	30% 1	30% 1	40% 1	50% 2	70% 4	0% 0	0% 0	30% 2	20% 1	50% 3	0% −	10% 1	10% 1	40% 3
b) *A. junnii*(2)	2	1	2	0	0	0	1	0	1	0	0	−	1	2	−	−	−	0
c)14TU(1)	−	−	1	−	−	−	−	1	−	−	−	−	−	−	−	−	−	0
d) *A. variabilis* (2)	1	1	1	1	1	1	1	1	1	0	0	−	−	−	−	−	−	1
e) *A. haemolyticus*(1)	1	1	1	1	1	1	1	1	1	0	0	1	−	−	−	−	−	0
*P*-value	0.96012	0.68916	0.65272	0.04036	0.14156	0.14156	0.1556	0.12114	0.19706	0.67448	–	–	–	–	–	–	–	–

*CAZ, Ceftazidime; FEP, Cefepime; AZT, Aztreonam; DOR, Doripenem; IMP, Imipenem; MEM, Meropenem; AN, Amikacin; GEN, Gentamicin; CIP, Ciprofloxacin; MIN, Minocycline; TGC, Tigecycline*.

*Significant p-value is underlined*.

The identity of the isolates using the ARDRA method was compared with Mini API or VITEK 2 results. Twenty six of the 38 strains were identified as the same species when results of the mini API and ARDRA (Table S2) were compared. Similarly, 19 of the 30 isolates were assigned as the same species by VITEK 2 and ARDRA (Table S2).

### Antimicrobial susceptibility tested by VITEK 2

Table 1 summarizes the antibiotic resistance pattern found in *Acinetobacter* spp. during this period. More than 75% isolates were resistant to extended spectrum cephalosporins (79% isolates were resistant to ceftazidime, 75% isolates were resistant to cefepime), 85% isolates were resistant to aztreonam, 50% were resistant to doripenem, 51% were resistant to both imipenem and meropenem. More than 60% isolates were resistant to at least one aminoglycosides (60% resistant to amikacin, 71% resistant to gentamicin). Eighty four percent isolates were resistant to ciprofloxacin. But all isolates showed high susceptibility to minocycline (99%) and tigecycline (100%).

All the *Acinetobacter* isolates were categorized into two groups, the ACB complex (*A. baumannii, A. calcoaceticus, A. nosocomialis*, and *A. pittii*) and other species (*A. lwoffii, A. junni, A. haemolyticus, A. variabilis*, and 14TU). Several analyses in this study were done where comparison between these two groups were made.

While analyzing resistance to different groups of antibiotics against these two categories, differences were noted for doripenem (Table 1). The percentage of doripenem-resistant isolates was significantly higher in ACB complex (55%) than in other species (20%) (*p*-value 0.0404). However, since the number of strains in the category “other species” is small (*n* = 10), these differences should not be overemphasized.

### Distribution of MIC values of different groups of antibiotics

There was a marked increase in resistance to carbapenems among *Acinetobacter* spp. from 25% in 2007 to 87% in 2013 (Figure S1). The range of MIC values for meropenem and ceftazidime over this period were 0.025− ≥32 mg/L and 1− ≥256 mg/L respectively. For individual years, the range of MIC values is depicted in Table S3.

Over this period the MIC_50_ and MIC_90_ values for meropenem were 32 mg/L and for ceftazidime were 256 mg/L. Detailed year-wise distribution of MIC_50_ and MIC_90_ values (mg/L) for meropenem and ceftazidime is shown in Table S3. This result depicted a significant increase in MIC value of meropenem since 2011 whereas for ceftazidime high resistance persisted throughout this period (except for 2010) (Table S3).

### Detection of different β-lactamases by phenotypic test

Of the 68 isolates, 19, 24, and 28% showed phenotypic evidence of MBLs, ESBLs and AmpCs respectively. Among MBL-producers (*n* = 13), 50% isolates showed ESBL and AmpC phenotype both and 64% showed only ESBL phenotype. Whereas, among non-MBL (*n* = 55) phenotype, 18% were ESBL producers, 27% were AmpC producers and 11% produced both of the enzymes. Presence of MBL sometimes complicates the detection of ESBLs and AmpCs. Therefore, the presence of ESBLs and AmpCs in these isolates was confirmed by PCR subsequently.

### Genotypic distribution of various β-lactamases

Isolates possessed different β-lactamases such as ESBLs (50%), AmpC (52%), and carbapenemases (56%, includes both oxacillinases and MBLs). Sequencing revealed the predominant presence of *bla*_PER−1_(35%), followed by *bla*_VEB−2_ and *bla*_CTXM−15_. Among the carbapenem-resistant determinants the *bla*_OXA−23−like_ gene (49%) was the most common, followed by *bla*_OXA−58−like_ gene (15%). Sequence analysis revealed that NDM-1 was the predominant (22%) MBL detected (Table 1). No other variant of NDM was found.

Detailed distribution of these genes in the different species has been presented in Table 1. *bla*_NDM−1_ was present in 21% of the ACB complex (*A. calcoaceticus, A. baumannii, A. nosocomialis*) and 30% of the other species (*A. haemolyticus, A. lwoffii*). In contrast, most of the oxacillinases were confined within ACB complex except 2 isolates of *A. lwoffii* carried either *bla*_OXA−23−like_ or *bla*_OXA−58−like_ gene.

While correlating the phenotypic and genotypic test results of all the strains, it was observed that one strain that could not be detected as MBL producers by phenotypic tests harbored *bla*_NDM−1_ (A_134) as detected by PCR. Similarly, there were 8 strains which did not show the presence of ESBL by the phenotypic tests but harbored *bla*_PER−1_ or *bla*_CTXM−15_. Of these 8 strains, 3 showed the presence of NDM-1. Similarly, 17 of the AmpC non-producers (as tested by the phenotypic tests) harbored *bla*_AmpC_ gene and 5 of these strains also possessed NDM-1. This showed the failure to detect ESBLs and AmpCs by phenotypic tests particularly in MBL–producing strains.

### Diversity of the *Acinetobacter* isolates

**Figure 1:**
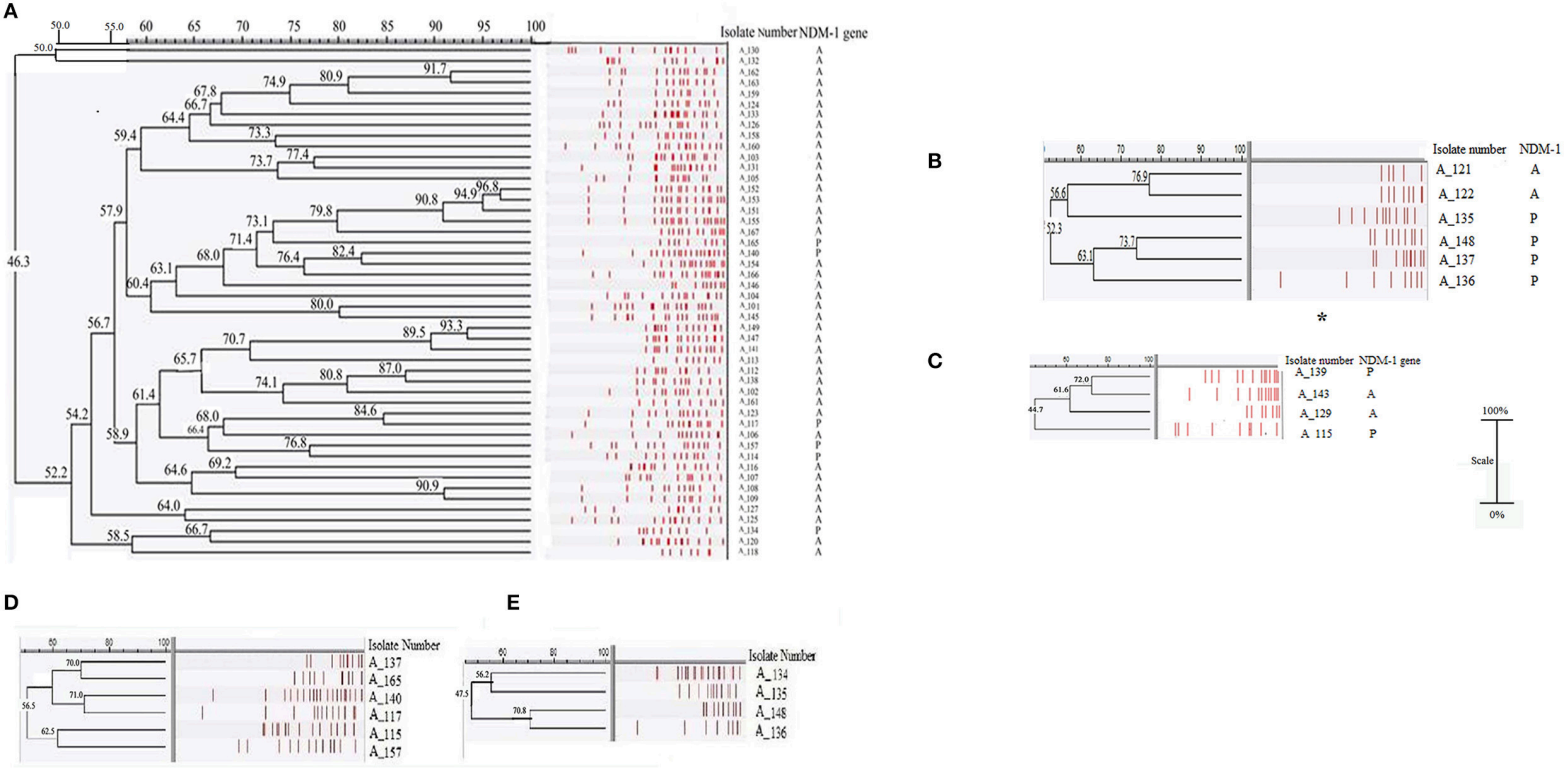
**Analysis of genetic relationship according to Dice's similarity coefficient and the unweighted pair group method with arithmetic mean (UPGMA) (the position tolerance and optimization were set at 1.5 and 1% respectively) of the ApaI patterns of (A) ***A. baumannii*** (***n*** = 49), (B) ***A. calcoaceticus*** (***n*** = 6), (C) ***A. lwoffii*** (***n*** = 4) isolates, (D) ***A. baumannii*** (***n*** = 6), and (E) ***A. calcoaceticus*** (***n*** = 4) harboring NDM-1**. More than 90% similarity in PFGE band pattern interpreted as indistinguishable. A few pairs of strains were found to be indistinguishable but could not be considered as episodes of cross-transmission as they were isolated from neonates who were not treated in the NICU during an interval of 15 days: (i) A_152 (DOB 06/01/2013) and A_153 (DOB 24/01/2013), (ii) A_162 (DOB 5/09/2013) and A_163 (DOB 21/10/2013), (iii) A_151 (DOB 10/01/2013) and A_155 (DOB 08/03/2013), (iv) A_108 (DOB 24/08/2008) and A_109 (DOB 24/11/2008), (v) A_147 (DOB 25/01/2012) and A_149 (DOB 01/04/2012). ^*^One *A. calcoaceticus* isolate was untypable. A represents absence of NDM-1, P represents presence of NDM-1.

The analyses of the pulsotypes were done separately for *A. baumannii, A. calcoaceticus*, and *A. lwoffii*. Strains of *A. baumannii* were primarily diverse and dissemination of any particular clone was not evident (Figure 1). One sporadic case of cross-transmission was seen: A_151 (DOB 10/01/2013) and A_153 (DOB 24/01/2013). A few pairs of strains were found to be indistinguishable but could not be considered as episodes of cross-transmission as they were isolated from neonates who were not treated in the NICU during an interval of 15 days (Figure 1). In *A. calcoaceticus* and *A. lwoffii* group no cross-transmission was noted and all the isolates were distinct.

The number of isolates in other groups (*A. junni, A. nosocomialis*, 14TU, *A. variabilis, A. haemolyticus*) was small for cluster analysis and were visually analyzed. All were diverse.

### Characterization of the *bla*_NDM−1_-harboring isolates

Twenty two percent (*n* = 15) of the isolates harbored NDM-1 across all species of *Acinetobacter*. The presence of NDM-1 was not significantly different between the ACB complex and the other species (*p*-value: 0.7640). Most of the isolates (*n* = 11) harboring NDM-1 had an MIC value ≥32 mg/L for meropenem except for a few that had low MIC values (0.125, 0.25, 0.5, 1 mg/L) (Table 2). One of the isolates, A_117 (0.5 mg/L) had at least three carbapenem resistant determinants *bla*_NDM−1_, *bla*_OXA−23−like_ and *bla*_VIM−2_gene. All NDM-1-harboring isolates were resistant to a series of antibiotics except tigecycline and colistin.

**Table 2:** **Characterization of NDM-1-harboring ***Acinetobacter*** spp**.

**Isolate No**.	**Molecular**	**MIC values (mg/L)**												**Genetic determinants**	**No. of plasmid/plasmid**
	**Identification**	**MEM**	**IMP**	**DOR**	**CAZ**	**FEP**	**AZT**	**AN**	**GEN**	**CIP**	**MIN**	**TGC**	**COL**		**sizes kb (approx.)**
A_114	*A. lwoffii*	≥16	8	0.5	≥64	≥64	≥64	≥64	≥16	≥4	≤ 1	≤ 0.5	1	*bla*_NDM−1_, *bla*_OXA58_, *bla*_AmpC_, *bla*_CTX−M−15_,*IntI1*.	3/119, 5, 2
A_115	*A. baumannii*	8	16	8	≥64	≥64	16	16	4	≥4	≤ 1	≤ 0.5	≤ 0.5	*bla*_NDM−1_, *bla*_OXA51_, *bla*_AmpC_, *aac(6′)-Ib-cr*.	1/122
A_117	*A. baumannii*	0.5	≤ 0.25	0.25	≤ 1	≤ 1	2	≥64	8	2	≤ 1	≤ 0.5	≤ 0.5	*bla*_NDM−1_,*bla*_VIM−2_,*bla*_OXA51, 23_, *bla*_AmpC_, *IntI1*.	4/82, 71, 44, 16
A_134	*A. calcoaceticus*	≥16	≥16	≥8	≥64	16	≥64	≥64	≥16	≥4	≤ 1	2	≤ 0.5	*bla_NDM_*_−1_, *bla_OXA_*_51, 23_,*bla*_AmpC_, *armA*,*bla*_PER−1_, *IntI1*.	1/208
A_135	*A. calcoaceticus*	≥16	≥16	≥8	≥64	16	16	16	≥16	≥4	≤ 1	≤ 0.5	≤ 0.5	*bla*_NDM−1_, *bla*_PER−1_,*IntI2*.	1/213
A_136	*A. calcoaceticus*	≥16	≥16	≥8	≥64	2	16	16	≥16	2	≤ 1	≤ 0.5	≤ 0.5	*bla*_NDM−1_, *bla*_OXA58_,*bla*_AmpC_, *armA, bla*_PER−1_	3/161,5,3
A_137	*A. baumannii*	≥16	≥16	≥8	≥64	≥64	4	≤ 2	≥16	≥4	≤ 1	≤ 0.5	≤ 0.5	*bla*_NDM−1_,*armA, bla*_PER−1_,*aac*(6′)-*Ib*,*IntI1*.	1/117
A_139	*A. lwoffii*	1	0.5	≤ 0.25	≥64	≥64	≥64	2	≤ 1	≥4	≤ 1	≤ 0.5	≤ 0.5	*bla*_NDM−1_,*bla*_VEB−2_.	1/120
A_140	*A. baumannii*	≥16	≥16	≥8	≥64	≥64	≥64	32	≥16	≥4	≤ 1	≤ 0.5	≤ 0.5	*bla*_NDM−1_, *bla*_OXA51, 23_,*bla*_AmpC_, *armA,aac(6′)-Ib,IntI2*.	1/120
A_148	*A. calcoaceticus*	0.125	≤ 0.25	≤ 0.12	≥64	32	≥64	16	≤ 1	≥4	≤ 1	≤ 0.5	≤ 0.5	*bla*_NDM−1_,*aac(6′)-Ib, bla*_PER−1_, *IntI1*.	1/99
A_156	*A. nosocomialis*	≥16	≥16	≥8	≥64	≥64	≥64	≥64	≥16	≥4	≤ 1	1	≤ 0.5	*bla*_NDM−1_,*bla*_OXA58_, *bla*_AmpC_, *aac(6′)-Ib, bla*_PER−1_, *IntI1*.	1/88
A_157	*A. baumannii*	0.25	0.5	0.25	≥64	≥64	≥64	≥64	≥16	≥4	≤ 1	≤ 0.5	≤ 0.5	*bla*_NDM−1_, *bla*_OXA51, 58_, *armA, bla*_PER−1_, *IntI1*.	1/86
A_164	*A. nosocomialis*	≥16	8	≥8	≥64	32	2	16	≤ 1	1	≤ 1	≤ 0.5	≤ 0.5	*bla*_NDM−1_,*bla*_OXA23_,*bla*_VEB−2_, *bla*_AmpC_.	1/204
A_165	*A. baumannii*	≥16	≥16	≥8	≥64	≥64	≥64	≥64	≥16	≥4	≤ 1	≤ 0.5	≤ 0.5	*bla*_NDM−1_, *bla*_OXA51, 23_,*bla*_VEB−2_,*bla*_AmpC_, *armA, IntI1*.	1/207
A_168	*A. haemolyticus*	≥16	≥16	≥8	≥64	≥64	≥64	≥64	≥16	≥4	≤ 1	1	≤ 0.5	*bla*_NDM−1_,*armA, IntI1*.	1/101

Most isolates that possessed *bla*_NDM−1_ also possessed *bla*_OXA−23−like_ or *bla*_OXA−58−like_. NDM-1 possessing isolates also harbored *bla*_VIM−2_ (6%)′ *bla*_VEB−2_ (20%), *bla*_PER−1_ (60%), *bla*_AmpC_ (60%), *armA* (47%), *aac(6*′*)-Ib* (27%). Presence of class 1 integron was observed in 8 of the NDM-1-possessing isolates; *IntI2* was present in one isolate (Table 2).

PFGE of the *A. baumannii* and *A. calcoaceticus* isolates possessing NDM-1 revealed that diverse clones of these species harbored NDM-1(Figure 1). No cases of cross-transmission were noted in the NDM-1 isolates.,

### Characterization of the *bla*_NDM−1_-harboring transconjugants

Fourteen of the 15 NDM-1 harboring isolates were able to transfer *bla*_NDM−1_ by conjugation (13/15) or transformation (1/15) to the recipients. Fifty percent of the transconjugants showed MIC values of ≥3 mg/L for meropenem and 79% of the isolates were also resistant to ceftazidime (MICs ≥ 8 mg/L). One transformant (A_139-DH10B/EC) was sensitive to both meropenem (0.023 mg/L) and ceftazidime (0.5 mg/L) (Table 3). A single plasmid was detected in all of the *bla*_NDM−1_-harboring transconjugants and the sizes of these plasmids ranged between 86 and 213 kb (Table 3).

**Table 3:** **Detailed characterization of the transconjugants harboring NDM-1**.

**Transconjugant/Transformant numbers**	**MIC values(mg/L)**		**Gene profiles**	**5′-3′conserved element (bp approx.)/In numbers**	**Linkage with different IS elements**	**No. of plasmid/plasmid sizes kb (approx.)**
	**MEM**	**CAZ**				
A_114-J53	3	≥256	*bla*_NDM−1_, *bla*_OXA−96_.	–	TnAba125-*bla*_NDM1_, *IS*Aba3-*bla*_OXA−96_	1/119
A_115-J53	16	≥256	*bla*_NDM−1_, *aac(6′)-Ib-cr*.	–	TnAba125-*bla*_NDM−1_	1/122
A_134-J53	0.38	≥256	*bla*_NDM−1_, *bla*_OXA−73_, *armA, bla*_PER−1_, *IntI1*.	5′CS- *aadB*- *gcuE2*-*gcu8*-*cmlA1g*- 3′CS (2900)/In571	TnAba125-*bla*_NDM−1_, *IS*Aba1-*bla*_OXA−73_	1/208
A_135-J53	8	≥256	*bla*_NDM−1_,*bla*_PER−1_,*IntI2*	–	*IS*Aba125-*bla*_NDM−1_	1/213
A_136-J53	16	≥256	*bla*_NDM−1_,*bla*_OXA−164_,*armA, bla*_PER−1_	–	TnAba125-*bla*_NDM−1_, *IS*Aba3-*bla*_OXA−164_	1/161
A_137-J53	0.064	≥256	*bla*_NDM−1_, *aac(6′)-Ib, armA, bla*_PER−1_, *IntI1*.	5′CS- *aadB*- *gcuE2*-*gcu8*-*cmlA1g*- 3′CS (2900)/In571	TnAba125-*bla*_NDM−1_	1/117
A_139-DH10B/EC-Transformant	0.023	0.5	*bla*_NDM−1_	–	–	1/120
A_140-J53	32	8	*bla*_NDM−1_, *bla*_OXA−225_, *aac(6* ′*)-Ib, armA, IntI1*.	5′CS-*dfrA12*- *gcuF*-*aadA2*-3′CS (2000)/In27	TnAba125-*bla*_NDM−1_, *IS*Aba1-*bla*_OXA−225_	1/120
A_148-J53	0.064	≥256	*bla*_NDM−1_, *bla*_OXA−171_, *aac(6*′*)-Ib, bla*_PER−1_, *IntI1*.	5′CS-*arr3-aacA4*-3′CS (1500)/In311	TnAba125-*bla*_NDM−1_, *IS*Aba1-*bla*_OXA−171_	1/99
A_156-J53	6	≥256	*bla*_NDM−1_, *bla*_OXA−96_, *aac(6*″*)-Ib, IntI1*	5′CS- *aadB*- *gcuE2*- *gcu8*- *cmlA1g*- 3′CS (2900)/In571	TnAba125-*bla*_NDM−1_, *ISAba3*-*bla*_OXA−96_	1/88
A_157-J53	0.064	12	*bla*_NDM−1_, *armA, IntI1*	5′CS-*blaCARB−2*- 3′CS (1117)/In167	TnAba125-*bla*_NDM−1_	1/86
A_164-J53	3	≥256	*bla*_NDM−1_, *bla*_VEB−2_.	–	TnAba125-*bla*_NDM−1_	1/204
A_165-J53	0.38	8	*bla*_NDM−1_, *bla*_VEB−2_, *armA, IntI1*.	5′CS-*dfrA12*- *gcuF*-*aadA2*-3′CS (1835)/In27	TnAba125-*bla*_NDM−1_	1/207
A_168-J53	0.094	≥256	*bla*_NDM−1_, *armA, IntI1*	5′CS- *arr2- cmlA1g*-3′CS (2305)/In310	TnAba125-*bla*_NDM−1_	1/101

**Figure 2:**
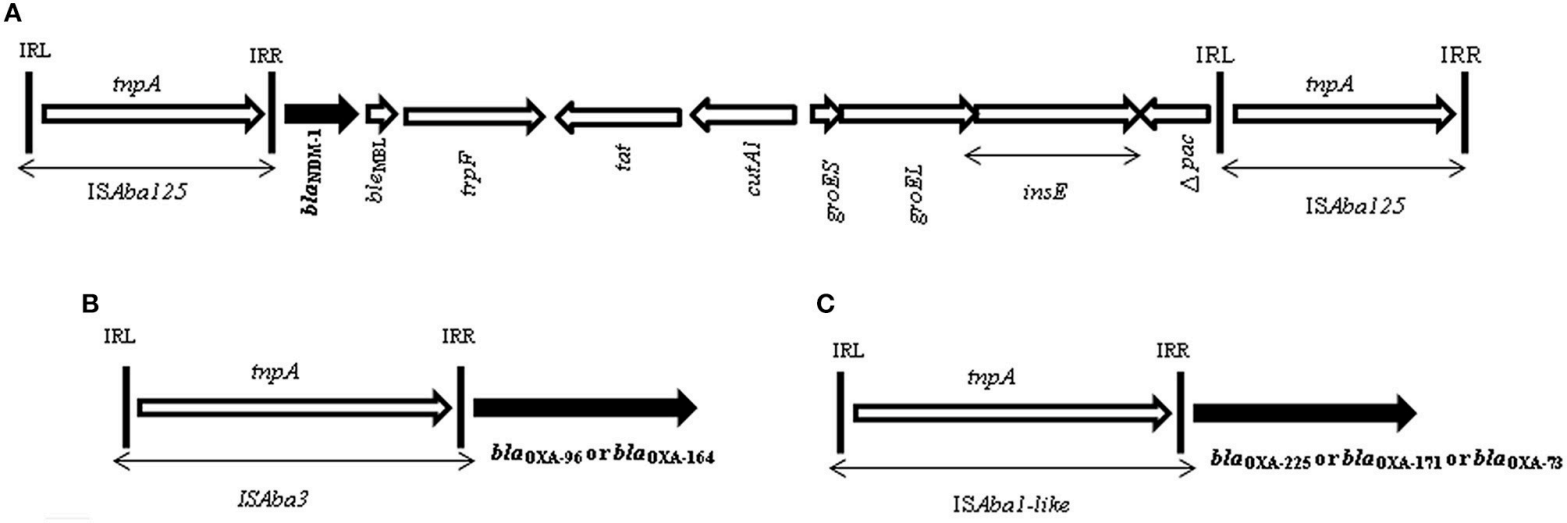
**(A)** Schematic representation of Tn125 carrying *bla*_NDM−1_ gene in a representative transconjugant. Genes and their transcription orientation are indicated by arrows. The lengths of the target genes and their exact location of the target genes are not to scale. Gene names are abbreviated according to the corresponding proteins (Bonnin et al., 2012): *cutA1* for divalent cation tolerence protein; *groES, groEL* for heat-chaperonin protein; *insE* for *IS*CR21 of tnpA family. Δ *pac* for truncated phospholipid acetyltransferase. IRL and IRR are for inverted repeat left and right, respectively. This structure was found in all of the transcojugants (except one transformant). **(B)** Diagram showing the linkage between *bla*_OXA−58−like_ with *IS*Aba3 (A_114-J53, A_136-J53, and A_156-J53). **(C)** Diagram showing the linkage between *bla*_oxa−23−like_ with *IS*Aba1 (A_134-J53, A_140-J53, and A_148-J53).

Investigation of the immediate genetic environment of *bla*_NDM−1_ gene revealed the presence of a conserved structure that always associates with the complete transposon, Tn125. This structure starts with one copy of the *IS*Aba125 at the 5′ end of *bla*_NDM−1_ gene and *ble*_MBL_ gene followed by *trpF, tat, cutA1, groES, groEL, insE*, and *IS*Aba125 at the 3′ end. All strains showed similar genetic structure surrounding the *bla*_NDM−1_. As the primer walking and PCR results were consistent for all transconjugants, sequences were thoroughly analyzed for 4 isolates (Figure 2).

PCRs were also carried out to map the position of the respective *IS* elements with the *bla*_OXA−23−like_ and *bla*_OXA−58−like_ genes in the transconjugants harboring NDM-1. Among the NDM-1-harboring transconjugants, three possessed *bla*_OXA−23−like_ and three others harbored *bla*_OXA−58−like_ gene (Table 3). All transconjugants with *bla*_OXA−23−like_were linked to *IS*Aba1 and all three isolates with *bla*_OXA−58−like_were linked to *IS*Aba3 (Figure 2). Sequence analysis revealed the presence of different variants of OXA-23-like enzymes, i.e., OXA-73 (A_134), OXA-225 (A_140), and OXA-171(A_148). Similarly, *bla*_OXA−58−like_ genes were identified as OXA-96 (A_114, A_156) and OXA-164 (A_136) (Table 3).

**Figure 3:**
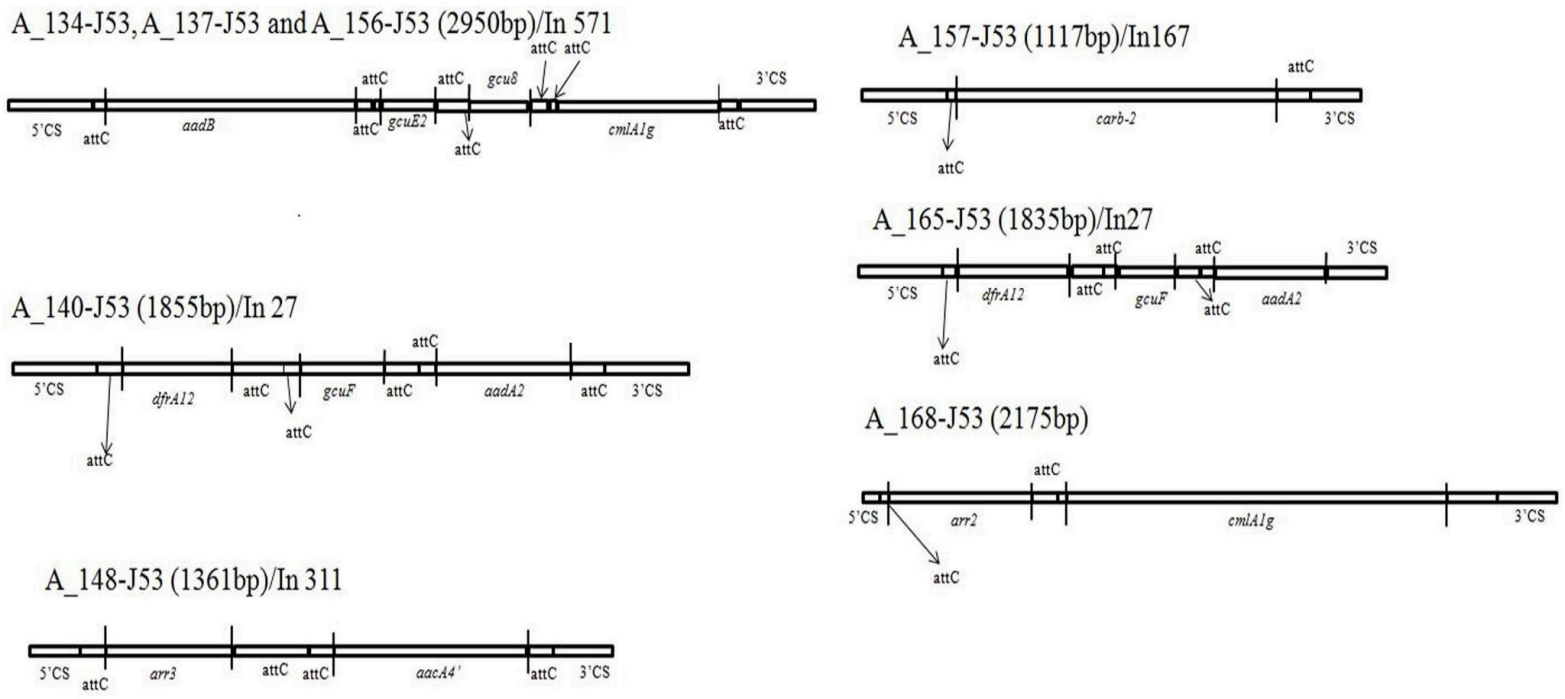
**Schematic diagram of genes in between 5′CS and 3′CS of class 1 integron of 8 transconjugants of ***Acinetobacter*** spp**. Three of the transconjugants (A_134-J53, A_137-J53, and A_156-J53) share similar structure. The genes are not to scale and vertical lines represent the boundaries of each gene. Gene names are abbreviated according to their corresponding proteins: *aadB, aadA2, aacA4*′− resistance to trimethoprim, *gcuF, gcuE2, gcu8*- unknown ORF, *arr3*, and *arr2*- ADP-ribosylation gene, *carb-2*- resistance to β-lactamases. attC site- attachment sites of each gene.

Analysis of the integrons revealed different combinations of resistance genes bracketed between the 5′ and 3′ conserved region and each of these different resistance gene cassettes are presented in Figure 3. Most commonly found genes in this conserved segment were aminoglycoside resistance (*aadB, aadA2, aac4*′), non-enzymatic chloramphenicol resistance gene (*cmlA1g*) and ADP-ribosylation gene (*arr2, arr3*).

### Association of clinical factors and mortality of the neonates with the presence of NDM-1-harboring *Acinetobacter*

The results of a comparison of neonates with sepsis caused due to NDM-1-harboring *Acinetobacter* with those without NDM-1 is shown in Table 4. Multivariate logistic regression established that neonates who were outborn had a significantly higher incidence of NDM-1 harboring isolates than their inborn counterparts [Odds Ratio (OR) 2.39; *p*-value <0.001]. None of the other factors were found to be statistically significant.

**Table 4:** **Association of clinical factors with sepsis caused by NDM-1-harboring ***Acinetobacter*** spp. in neonates**.

**Clinical factors**		**Neonates with NDM-1 (*****n*** = **15)^$^**		**Neonates without NDM-1 (*****n*** = **53)^#^**		***P*-value**
**No. of Neonates**		***n***	**%**	***n***	**%**	
Sex	Male	8	18.2	36	81.6	–
	Female	5	27.8	13	72.7	
Gestational Age	Pre-term	11	20.8	42	79.2	–
	Term	2	22.2	7	77.8	
Inborn/ Outborn	Inborn	7	16.3	36	83.7	>0.001^**^
	Outborn	6	31.6	13	68.4	
Birth Weight	Low birth weight	10	20	40	80	–
	Normal birth weight	3	25	9	75	
Baby on Ventilation	Yes	8	20	32	80	–
	No	5	22.7	17	77.3	
Mode of Delivery	Caesarean delivery	8	19.5	33	80.5	–
	Normal vaginal delivery	5	23.8	16	76.2	

*^#^No clinical data was available for four patients*.

** *Significant at 95% confidence*.

$ *No clinical data was available for two patients*.

Incidence of NDM-1 in late onset sepsis cases (38.1%) was significantly higher than those in early onset sepsis (12.2%) [OR 4.43; *p* < 0.0177].Finally, the association analysis between NDM-1-harboring *Acinetobacter* isolates and mortality (18.8% in death cases vs. 21.7% in discharge cases), showed no statistically significant difference [*p*-value 0.8003].

## Discussion

The data show that the potential impact of *Acinetobacter* sepsis on neonates in a tertiary care center is significant. There has been an increase of sepsis due to lactose non-fermenting Gram-negative bacteria in recent years and *Acinetobacter* is an important contributor to this burden (Viswanathan et al., 2014). This study also shows that though *A. baumannii* is still by far the most common of the *Acinetobacter* species causing sepsis in this unit, other species are also emerging in the clinical settings. Though infections due to other species of *Acinetobacter* has been reported (Turton et al., 2011; Tien et al., 2012), infection in the neonatal population caused by species of *Acinetobacter* other than *A. baumannii* are rare. The use of the ARDRA method in this study has helped in the elucidation of the species providing an insight about the presence of the other species of *Acinetobacter* in cases of neonatal septicaemia.

Another issue that emerges from the study is drug resistance. Carbapenem resistance is high and the clinician is left with very little choice of antibiotics. The only antibiotic that still retains activity is tigecycline, an observation that has also been made by other authors (Golanbar et al., 2011; Hsieh et al., 2014). In this unit, piperacillin/tazobactum and amikacin are being used as a pre-emptive (first line) therapy for clinically suspected cases of sepsis. As a second line of therapy ofloxacin and amikacin are used in the unit. In rare cases where the above therapies are ineffective and the antibiogram results are still awaited, meropenem is used.

Further, this study also shows that the phenotypic detection of ESBLs and AmpCs in presence of MBLs is challenging. The failure to detect the ESBLs in the MBL–producing clinical isolates may lead to the hidden spread of such β-lactamases complicating the situation even further. The authors have recently devised a method to circumvent this problem (Datta et al., 2015).

Resistance to carbapenems and cephalosporins could be correlated to the diverse repertoire of genetic determinants that were identified. The most prevalent were the oxacillinases. Among the transmissible oxacillinases, OXA-23-like was the most common and this result is confirmatory of other studies (Woodford et al., 2006; Golanbar et al., 2011). The presence of the oxacillinases was significantly higher in the ACB complex compared to the other *Acinetobacter* species. The presence of ESBLs like PER-1, VEB-2 and CTXM-15 were also noted along with the carbapenemases in some isolates but compared to the carbapenemases their numbers were lower (Ben et al., 2011). Of the class B β-lactamases, only NDM-1 and VIM-2 were identified. Most earlier reports of NDM-1 or a variant of the gene in clinical settings have been in *A. baumannii* (Wang et al., 2007; Boulanger et al., 2012; Revathi et al., 2013) but the presence of NDM-1 in this study was noted across other species of *Acinetobacter* along with *A. baumannii*.

PFGE revealed that NDM-1 harboring isolates were diverse. *Acinetobacter* spp. possessing NDM-1 was isolated predominantly from neonates delivered at other (extramural) centers reflecting the dissemination of this gene but not any clone in particular. A study with *A. baumannii* isolates collected across Europe also reported diversity of clones in *Acinetobacter* infection (Bonnin et al., 2012). Studies that have reported the presence of NDM-1 in other genuses have also described similar diversity of clones (Pasteran et al., 2014).

The *bla*_NDM−1_ gene was organized in a composite transposon bracketed between two copies of *IS*Aba125 in all the isolates irrespective of the species. This arrangement of the gene in *Acinetobacter* has been reported earlier (Pfeifer et al., 2011; Bonnin et al., 2012; Fu et al., 2012). The similar organization of the *bla*_NDM−1_ in the diverse clones of *A. baumannii* and also the other species lends credence to the hypothesis that the current dissemination of the gene in *Acinetobacter* is likely linked to Tn125 and not plasmids. The linkage of *bla*_OXA−23−like_ and *bla*_OXA−58−like_ to *IS*Aba1 and *IS*Aba3 respectively as observed here, has also been reported in several earlier studies. This shows that the genetic context of these genes in *Acinetobacter* remain similar even in geographically distant places (Chen et al., 2010; Lin et al., 2010).

Analysis of the class I integron showed that though there was variability in the genetic determinants present between the 5′ and 3′ CS segments, most of the genes were not associated with carbapenem or cephalosporin resistance (except *carb-2*/A_157).

A few isolates harboring NDM-1 and oxacillinases had low MIC values (even as low as 0.125 mg/L in one isolate). Similar observation was also reported from earlier studies (Datta et al., 2014; Zmarlicka et al., 2015). Further work is needed to understand the reason for such differences which can occur due to alterations in the expression of the enzymes or other changes in the mechanism of resistance toward β-lactamases.

Statistical analysis indicated that a neonate born in other facilities (outborn) had a significantly higher incidence of NDM-1 harboring isolates than neonates born at this center. This indicates the possibility of the neonates having acquired NDM-1-harboring isolates from other hospitals where they were born. This is supported by the fact that most had a late onset of sepsis, the initial infection probably having acquired from the other hospitals. The clonal diversity of the isolates also lends support to this possibility.

This study documents the significance of both *Acinetobacter baumannii* and the other species, in cases of neonatal sepsis in a unit over a period of 7 years. We have been able to gain a unique assessment of the diverse genetic determinants responsible for carbapenem resistance in these isolates. The genetic context of the carbapenem-resistant genes also provides insight about the importance of the *IS*Aba125 in the spread of NDM-1 in *Acinetobacter*. The emergence of NDM-1 among an already existing repertoire of oxacillinases is a challenge for clinicians and microbiologists alike. As new resistance mechanisms constantly evolve, both laboratory detection systems and infection control measures need to be enhanced.

## Ethical approval

The study protocol was carefully reviewed and approved by the Institutional Ethics Committee of the National Institute of Cholera and Enteric Diseases (Indian Council of Medical Research) (No. A-1/2013-IEC, dated 10th January 2014). Individual informed consent was waived because this study used currently existing sample collected during the course of routine diagnosis of sepsis and did not pose any additional risks to the patients. The patient records/information was anonymized and de-identified prior to analysis.

## Author contributions

Conceived and designed the experiments: SB. Performed the experiments: SC, SD, SR. Analyzed the data: SC, SD, SR, LR, SB. Coordinated collection of specimens, maintenance of clinical data: AS, RV, TS. Contributed reagents/materials/analysis tools: AS, RV, TS, LR, SB. Contributed to the writing of the manuscript: SC, LR, SB.

## Funding

The study was supported by Indian Council of Medical Research (ICMR) intramural fund. SC and SD were supported by senior research fellowships from ICMR and SR received a postdoctoral fellowship from ICMR.

### Conflict of interest statement

The authors declare that the research was conducted in the absence of any commercial or financial relationships that could be construed as a potential conflict of interest.

## Supplementary material

Figure S1 **Distribution of ***Acinetobacter*** spp. from 2007 to 2014**. Isolates were collected for 6 months in 2012 and till June in 2014.

Table S1 **Primers used in this study**.

Table S2 **Comparison of identity of species by Mini API (a)/VITEK 2 compact (b) and ARDRA**.

Table S3 **Year wise distribution of MIC_**50**_, MIC_**90**_, and range of MIC values for meropenem and ceftazidime**.
