# Supplementary figures and images for: Carbapenem Resistance in Acinetobacter baumannii and Other Acinetobacter spp. Causing Neonatal Sepsis: Focus on NDM-1 and Its Linkage to ISAba125
Source: Front Microbiol. 2016 Aug 8;7:1126. doi: 10.3389/fmicb.2016.01126 (PMC4976090; doi:10.3389/fmicb.2016.01126)

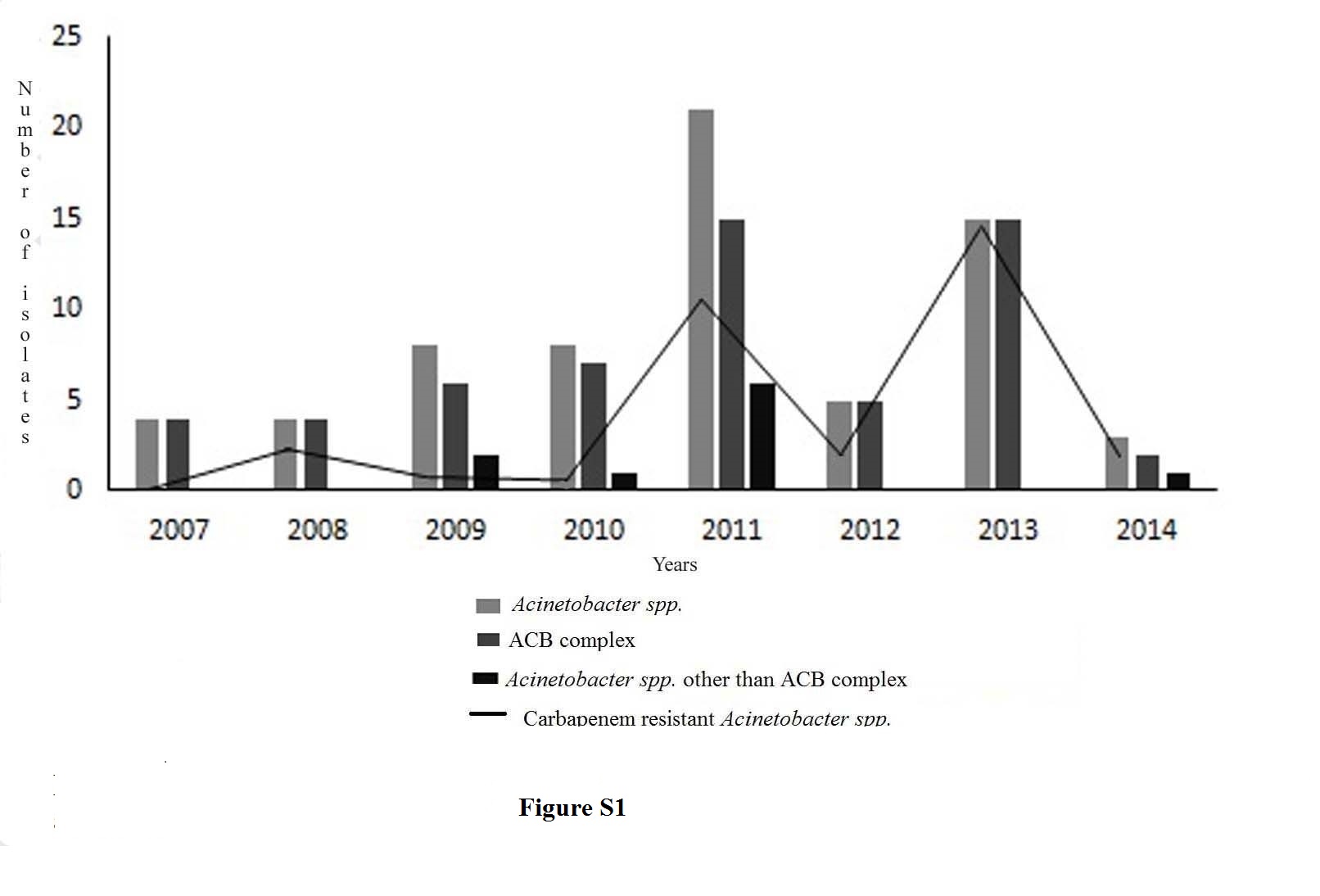

Supplement: Figure S1 — Distribution of Acinetobacter spp. from 2007 to 2014. Isolates were collected for 6 months in 2012 and till June in 2014. [file Image1.jpeg]
