# Supplementary material for: Carbapenem Resistance in Acinetobacter baumannii and Other Acinetobacter spp. Causing Neonatal Sepsis: Focus on NDM-1 and Its Linkage to ISAba125
Source: Front Microbiol. 2016 Aug 8;7:1126. doi: 10.3389/fmicb.2016.01126 (PMC4976090; doi:10.3389/fmicb.2016.01126)
Supplement: Table S1 — Primers used in this study. [file Table1.doc]

Supplementary table 1: Primers used in this study.

| PCR type | Name of the gene | Primer sequence (5'-3') | Amplified  product length (bp) | Reference |
| --- | --- | --- | --- | --- |
| Multiplex | OXA-51 | F-TAATGCTTTGATCGGCCTTG | 353 | Woodford *et al.*, 2006 |
|  |  | R-TGGATTGCACTTCATCTTGG |  |  |
|  | OXA-23 | F-ATTTCTGACCGCATTTCCAT | 501 |  |
|  |  | R-ATTCTGACCGCATTTCCAT |  |  |
|  | OXA-58 | F-AAGTATTGGGGCTTGTGCTG | 246 |  |
|  |  | R-CCCCTCTTGCGCTCTACATA |  |  |
|  | OXA-24 | F-GGTTAGTTGGCCCCCTTAAA | 599 |  |
|  |  | R-AGTTGAGCGAAAAGGGGATT |  |  |
|  | IMP | F-GGAATAGAGTGGCTTAAYTCTC | 188 | Mathews *et al.*, 2006 |
|  |  | R-CCA AACYACTA GTTAT T |  |  |
|  | VIM | F- GATGGTGTTTGGTCGCAT | 390 |  |
|  |  | R- CGA ATGCGCAGCACCAG |  |  |
|  | SIM | F- TACAAG GGATTCGGCATC | 570 |  |
|  |  | R- TAATGGCCTGTTCCCATGTG |  |  |
|  | GIM | F- TCG ACACACCTTGGTCTGAA | 477 |  |
|  |  | R- AACTTCCAACTTTGCCATGC |  |  |
|  | SPM | F- AAAATCTGGGTACGCAAACG | 271 |  |
|  |  | R- ACATTATCCGCTGGAACAGG |  |  |
| Singlex | 16S-23S rRNA | F- TGGCTCAGATTGAACGCTGGCGGC | 1500 | Dilkshoorn *et al.,* 1997 |
|  |  | R- TACCTTGTTACGACTTCACCCCA |  |  |
|  | NDM | F- GTCTGGCAGCACACTTCCTA | 515 | Roy *et al.,* 2011 |
|  |  | R- TAGTGCTCAGTGTCGGCATC |  |  |
|  | NDM-S | F- CCAATATTATGCACCCGGTCG | 813 | Espenal *et.al*, 2011 |
|  |  | R- ATGCGGGCCGTATGAGTGATTG |  |  |
|  | VEB | F- CGACTTCCATTTCCCGATGC | 689 | Cao *et al.*, 2002 |
|  |  | R- GGACTCTGCAACAAATACGC |  |  |
|  | VEB-S | F-GTTAGCGGTAATTTAACCAGATAG | 1071 | Jiang *et al.*, 2004 |
|  |  | R- CGGTTTGGGCTATGGGCAG |  |  |
|  | PER-S | F- ATGAATGTCATTATAAAAGCT | 953 | Perilli *et al.*, 2007 |
|  |  | R- TTACCAATGCTTAATCAGTGAG |  |  |
|  | Chromosomal  AmpC | F- ACTTACTTCAACTCGCGACG | 663 | Corvec *et.al*, 2003 |
|  |  | R- TAAACACCACATATGTTCCG |  |  |
|  | CTX-M  (consensus) | F- SCSATGTGCAGYACCAGTAA | 543 | Saladin  *et al.*, 2002 |
|  |  | R- CCGCRATATGRTTGGTGGTG |  |  |
|  | CTX-M-S | F-TTCGTCTCTTCCAGAATAAGG | 968 | Pfeifer *et al.*, 2009 |
|  |  | R-CAGCACTTTTGCCGTCTAAG |  |  |
|  | VIM-S | F-ATGTTCAAACTTTTGAGTAGTAAG | 748 | This study |
|  |  | R-CTACTCAACGACTGAGCG |  |  |
|  | ArmA | F-ATTCTGCCTATCCTAATTGG | 105 | Bercot *et al.,*2011 |
|  |  | R- ACCTATACTTTATCGTCGTC |  |  |
|  | AAC(6’)-IB | F-TTGCGATGCTCTATGAGTGGCTA | 482 | Kim *et.al*, 2009 |
|  |  | R-CTCGAATGCCTGGCGTGTTT |  |  |
|  | IntI1 | F-GCATCCTCGGTTTTCTGG | 457 | Shibata *et al.*, 2003 |
|  |  | R-GGTGTGGCGGGCTTCGTG |  |  |
|  | IntI2 | F-GGTGTGGCGGGCTTCGTG | 789 | Shibata *et al.*, 2003 |
|  |  | R-GTAGCAAACGAGTGACGAAATG |  |  |
|  | IntI3 | F-ATCTGCCAAACCTGACTG | 922 | Shibata *et al.*, 2003 |
|  |  | R-CGAATGCCCCAACAACTC |  |  |
|  | Integron conserved sequence | 5’CS CTTGCTGCTTGGATGCC | varied | Novais *et al*., 2006 |
|  |  | 3’CS AAGCAGACTTGACCTGAT |  |  |
|  | NDM-1 upstream | *IS*Aba125 ATGTATATTTCTGTGACCC | 915 | Poirel *et al.,*2011 |
|  |  | NDM L GACCGGGTGCATAATATTGG |  |  |
|  | NDM-1 downstream | NDM rt GATGCCGACACTGAGCACTA | 709 | Poirel *et al.,*2011 /This study |
|  |  | *ble*-rev GGCGATGACAGCATCATCCG |  |  |
|  |  | *ble*MBL-Trp F TGACAGAGGCGTTTTATGCG | 1009 | This study |
|  |  | bleMBL-Trp R CTTGTCGGTATCCTTGACGC |  |  |
|  |  | TrpF-CutA1F CGGGTGAAGTCGGGAAAATC | 1376 | This study |
|  |  | TrpF-CutA1R GACTTTCAAGGGCCGCAC |  |  |
|  |  | CutA1-groES F GGAGTTCGTAGGGATGCAGT | 709 | This study |
|  |  | CutA1-groES R TCGTCTTCCTTCACCACCAG |  |  |
|  |  | groES-EL F CTGCTAAAATCGCCGGGTTT | 1714 | This study |
|  |  | groES-EL R CGTGGTTCTGGTCTTCGTTC |  |  |
|  |  | groEL-insE F GTCGAAGGAAAACACCACCA | 1602 | This study |
|  |  | groEL-insE R TTGGCCAGTTCCGTCAGTT |  |  |
|  | OXA-23-like linkage | *IS*Aba1F/L GGATCCCTCTGTACACGAYAAATTTC | 855 | This study |
|  |  | OXA-23R/L GAATTCTTAAATAATATTCAGCTGTTTTAATG |  |  |
|  | OXA-58-like linkage | ISAba3 F/L CGTTTACCCCAAACATAAGC | 945 | This study |
|  |  | OXA-58 R/L TAACCTCAAACTTCTAATTC |  |  |
