# Supplementary material for: Carbapenem Resistance in Acinetobacter baumannii and Other Acinetobacter spp. Causing Neonatal Sepsis: Focus on NDM-1 and Its Linkage to ISAba125
Source: Front Microbiol. 2016 Aug 8;7:1126. doi: 10.3389/fmicb.2016.01126 (PMC4976090; doi:10.3389/fmicb.2016.01126)
Supplement: Table S2 — Comparison of identity of species by Mini API (a)/VITEK 2 compact (b) and ARDRA. [file Table2.doc]

Table S2: Comparison of identity of species by Mini API (a) / VITEK 2 compact (b) and ARDRA

a) Tested for isolates between 2007(Jan) to 2011(Aug)

| Identification  by ARDRA(n) | Mini API GN Result (n)^#^ |
| --- | --- |
| *A. baumannii* (27) | *A. baumannii* excellent identification (17), *A. baumannii* very good identification (2), *A. baumannii* good identification (2), *A. baumannii* low discrimination (3), *A. baumannii* doubtful profile (1), *A. lwoffii* (2) |
| *A. calcoaceticus* (6) | *A. lwoffii* excellent identification (2), *A. lwoffii* very good identification (2), *A. lwoffii* good identification (1), *A. lwoffii* low discrimination (1) |
| *A. lwoffii* (2) | *A. lwoffii* excellent identification (1), *A. junnii* very good identification (1) |
| *A. junni* (1) | *A. lwoffii* very good identification (1) |
| *A. variabilis* (2) | *A. lwoffii* excellent identification (1), *A.lwoffii* very good identification (1) |

b) Tested for isolates between 2011(Sep) to 2014(June)

| Identification  by ARDRA (n) | VITEK 2 Result (n)^#^ |
| --- | --- |
| *A. baumannii* (22) | ACB complex (18), *A. lwoffii* (3) non/low reaction biotype (1) |
| *A .calcoaceticus* (1) | *A. lwoffii*(1) |
| *A. nosocomialis* (2) | ACB complex (1), *A. lwoffii* (1) |
| *A. lwoffii* (2) | non/ low reaction biotype (1), unidentified (1) |
| *A. junni* (1) | Unidentified (1) |
| *A. haemolyticus* (1) | *A.lwoffii* (1) |
| 14TU (1) | *A.lwoffii* (1) |

#Isolates were tested either by Mini API or VITEK 2 compact system.
