# Supplementary material for: Carbapenem Resistance in Acinetobacter baumannii and Other Acinetobacter spp. Causing Neonatal Sepsis: Focus on NDM-1 and Its Linkage to ISAba125
Source: Front Microbiol. 2016 Aug 8;7:1126. doi: 10.3389/fmicb.2016.01126 (PMC4976090; doi:10.3389/fmicb.2016.01126)
Supplement: Table S3 — Year wise distribution of MIC50, MIC90, and range of MIC values for meropenem and ceftazidime. [file Table3.doc]

Table S3: Year wise distribution of MIC_50_, MIC_90_ and range of MIC values for meropenem and ceftazidime

The clinical breakpoints for meropenem were: susceptible (S) ≤2.0 mg/L, intermediate (I) 4.0 mg/L, and resistant (R) ≥8 mg/L and breakpoints for ceftazidime were: susceptible (S) ≤8.0 mg/L, intermediate (I) 16.0 mg/L, and resistant (R) ≥32 mg/L.

#Isolates were collected for 6 months in 2012 and 2014.

| Years (n) | Meropenem | | | Ceftazidime | | |
| --- | --- | --- | --- | --- | --- | --- |
|  | MIC_50_ | MIC_90_ | Range of MIC values (mg/L) | MIC_50_ | MIC_90_ | Range of MIC values (mg/L) |
| 2007(n=4) | 0.5 | 3 | 0.025-3 | 64 | ≥256 | 3-≥256 |
| 2008(n=4) | ≥32 | ≥32 | 0.25-≥32 | 64 | ≥256 | 64- ≥256 |
| 2009(n=8) | 0.38 | ≥32 | 0.125-≥32 | ≥256 | ≥256 | 16- ≥256 |
| 2010(n=8) | 0.25 | ≥32 | 0.047-≥32 | 2 | ≥256 | 1- ≥256 |
| 2011(n=22) | 3 | ≥32 | 0.125-≥32 | ≥256 | ≥256 | 1– ≥256 |
| 2012^#^(n=6) | ≥32 | ≥32 | 0.125-≥32 | ≥256 | ≥256 | ≥256 |
| 2013(n=15) | ≥32 | ≥32 | 0.25-≥32 | ≥256 | ≥256 | 4 - ≥256 |
| 2014^#^(n=3) | ≥32 | ≥32 | ≥32 | ≥256 | ≥256 | ≥256 |
